# Weak value amplification: a view from quantum estimation theory that highlights what it is and what isn’t

**DOI:** 10.1038/srep19702

**Published:** 2016-02-01

**Authors:** Juan P. Torres, Luis José Salazar-Serrano

**Affiliations:** 1 ICFO-Institut de Ciencies Fotoniques, The Barcelona Institute of Science and Technology, 08860 Castelldefels (Barcelona), Spain; 2 Dep. Signal Theory and Communications, Universitat Politècnica de Catalunya, 08034 Barcelona, Spain; 3 Quantum Optics Laboratory, Universidad de los Andes, AA 4976, Bogotá, Colombia

## Abstract

Weak value amplification (WVA) is a concept that has been extensively used in a myriad of applications with the aim of rendering measurable tiny changes of a variable of interest. In spite of this, there is still an on-going debate about its *true* nature and whether is really needed for achieving high sensitivity. Here we aim at helping to clarify the puzzle, using a specific example and some basic concepts from quantum estimation theory, highlighting what the use of the WVA concept can offer and what it can not. While WVA cannot be used to go beyond some fundamental sensitivity limits that arise from considering the full nature of the quantum states, WVA can notwithstanding enhance the sensitivity of *real and specific* detection schemes that are limited by many other things apart from the quantum nature of the states involved, i.e. *technical noise*. Importantly, it can do that in a straightforward and easily accessible manner.

Weak value amplification (WVA)^1^ is a concept that has been used under a great variety of experimental conditions^2^,^3^,^4^,^5^,^6^,^7^,^8^ to reveal tiny changes of a variable of interest. In all those cases, a priori sensitivity limits were not due to the quantum nature of the light used (*photon statistics*), but instead to the insufficient resolution of the detection system, what might be termed generally as *technical noise*. WVA was a feasible choice to go beyond this limitation. In spite of this extensive evidence, “its interpretation has historically been a subject of confusion”^9^. For instance, while some authors^10^ show that “weak-value-amplification techniques (which only use a small fraction of the photons) compare favorably with standard techniques (which use all of them)”, others^11^ claim that WVA “does not offer any fundamental metrological advantage”, or that WVA^12^ “does not perform better than standard statistical techniques for the tasks of single parameter estimation and signal detection”. However, these conclusions are criticized by some authors based on the idea that “the assumptions in their statistical analysis are irrelevant for realistic experimental situations”^13^. This can explain why recently some papers have appeared^10^,^14^ with the aim at *re-evaluating and re-considering* in a more general context what WVA can offer for metrology. Here we analyze from a new perspective (in terms of the quantum concept of trace distance), and by means of a particular example, but representative of a wide class of WVA experimental implementations, the pros and cons of using WVA, quantifying how much can be gained under appropriate circumstances. In other words, we will see, with an approach centered on how different quantum states are distinguished in specific experimental implementations, why some authors can say that WVA offers no advantage in metrology. At the same time we will put numbers to the idea that WVA can be *judged ‘handy’*^14^ in certain cases.

We use some simple, but fundamental, results from quantum estimation theory^15^ to show that there are two sides to consider when analyzing in which sense WVA can be useful. On the one hand, WVA measures specific information of a quantum state state obtained after transformations that in most cases make use of unitary operations. Therefore, it cannot modify the statistics of photons involved. Basic quantum estimation theory states that the post-selection of an appropriate output state obtained after unitary transformations, the basic element where WVA is embedded, cannot be better than the use of the input state^16^. Moreover, WVA uses some selected, useful but partial, information about the quantum state that cannot be better that considering the full state. Indeed, due to the unitarian nature of the operations involved in the overall transformation where WVA is embedded, it should be equally good any transformation of the input state than performing no transformation at all. In other words, when considering only the quantum nature of the light used, WVA cannot be expected to enhance the precision of measurements^17^.

On the other hand, a more general analysis that goes beyond only considering the quantum nature of the light, shows that WVA can be useful when certain technical limitations are considered (for instance, consider the examples presented in^10^,^13^). In this sense, it might increase the ultimate resolution of the detection system by effectively lowering the value of the smallest quantity that can detected. In most scenarios, although not always^18^, the signal detected is severely depleted, due to the quasi-orthogonality of the input and output states selected. However, in many applications, limitations are not related to the low intensity of the signal^2^, but to the smallest change that the detector can measure irrespectively of the intensity level of the signal.

A potential advantage of our approach is that we make use of the concept of trace distance, a clear and direct measure of the degree of distinguishability of two quantum states. Indeed, the trace distance gives us the minimum probability of error of distinguishing two quantum states that can be achieved under the best detection system one can imagine^15^. Measuring tiny quantities is essentially equivalent to distinguishing between nearly parallel quantum states. Therefore we offer a very basic and physical understanding of how WVA works, based on the idea of how WVA schemes transform very close quantum states, which can be useful to the general physics reader.

The approach used here is slightly different from what other analysis of WVA do, where most of the times the tool used to estimate its usefulness is the Fisher information and the related Cramér-Rao bound. The Fisher information requires to know the probability distribution of possible experimental outcomes for a given value of the variable of interest. Therefore, it can look for sensitivity bounds for measurements by including *technical characteristics* of specific detection schemes^10^. A brief comparison between both approaches will be done towards the end of this paper.

Some words of caution will be useful here. Firstly, for the sake of clarity, we make use of a specific WVA scheme aimed at measuring ultra-small temporal delays^19^ that has been recently demonstrated experimentally^8^,^20^. However, the scheme considered here is representative of a long list of *fundamentally equivalent* WVA schemes. Therefore, some of our conclusions might not apply to all WVA schemes without further consideration. We note that the use of a specific experimental configuration where WVA has demonstrated its *practical* utility in the lab, providing an enhancement in sensitivity, can help the general reader to identify more clearly the role of important concepts of our discussion such as quantum distance or technical noise.

Secondly, the concept of weak value amplification is presented for the most part in the framework of Quantum theory, where it was born. It can be readily understood in terms of constructive and destructive interference between probability amplitudes^21^. Interference is a fundamental concept in any theory based on waves, such as classical electromagnetism. Therefore, the concept of weak value amplification can also be described in many scenarios in terms of interference of classical waves^22^. Indeed, most of the experimental implementations of the concept, since its first demonstration in 1991^23^, belong to this type and can be understood without resorting to a quantum theory formalism.

## An example of the application of the weak value amplification concept: measuring small temporal delays with large bandwidth pulses

**Figure 1:**
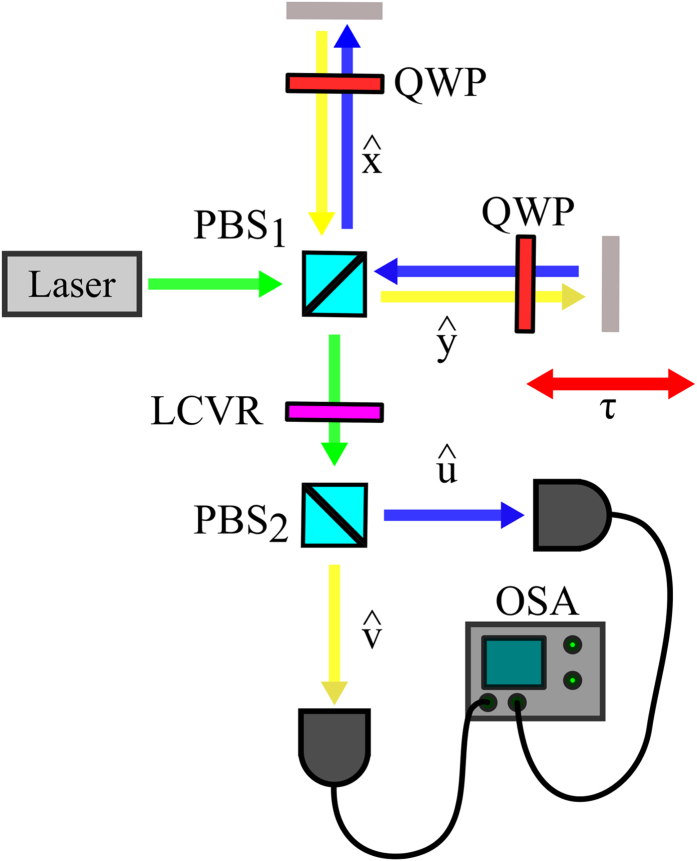
Weak value amplification scheme aimed at detecting extremely small temporal delays. The input pulse polarisation state is selected to be left-circular by using a polariser, a quarter-wave plate (QWP) and a half-wave plate (HWP). A first polarising beam splitter (PBS_1_) splits the input into two orthogonal linear polarisations that propagate along different arms of the interferometer. An additional QWP is introduced in each arm so that after traversing the QWP twice, before and after reflection in the mirrors, the beam polarisation is rotated by 90^°^ to allow the recombination of both beams, delayed by a temporal delay *τ*, in a single beam by the same PBS. After PBS_1_, the output polarisation state is selected with a liquid crystal variable retarder (LCVR) followed by a second polarising beam splitter (PBS_2_). The variable retarder is used to set the parameter *θ* experimentally. Finally, the spectrum of each output beam is measured using an optical spectrum analyzer (OSA). (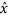, 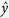) and (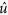, 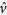) correspond to two sets of orthogonal polarisations.

For the sake of example, we consider a specific weak amplification scheme^19^, depicted in Fig. 1, which has been recently demonstrated experimentally^8^,^20^. It aims at measuring very small temporal delays *τ*, or correspondingly tiny phase changes^24^, with the help of optical pulses of much larger duration. We consider this specific case because it contains the main ingredients of a typical WVA scheme, explained below, and it allows to derive analytical expressions of all quantities involved, which facilitates the analysis of main results. Moreover, the scheme makes use of linear optics elements only and also works with large-bandwidth partially-coherent light^25^.

In general, a WVA scheme requires three main ingredients: a) the consideration of two subsystems (here two degrees of freedom: the polarisation and the spectrum of an optical pulse) that are weakly coupled (here we make use of a polarisation-dependent temporal delay that is introduced with the help of a Michelson interferometer); b) the *pre-selection* of the input state of both subsystems; and c) the *post-selection* of the state in one of the subsystems (the state of polarisation) and the measurement of the state of the remaining subsystem (the spectrum of the pulse). With appropriate *pre-* and *post-selection* of the polarisation of the output light, tiny changes of the temporal delay *τ* can cause anomalously large changes of its spectrum, rendering in principle detectable very small temporal delays.

Let us be more specific about how all these ingredients are realized in the scheme depicted in Fig. 1. An input coherent laser beam (mean number of photons, *N*) shows circular polarization, 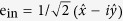, and a Gaussian shape with temporal width *T*_0_ (Full-width-half maximum, *τ* ≪ *T*_0_). The normalized temporal and spectral shapes of the pulse read


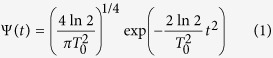



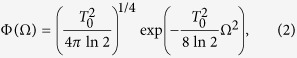


where *t* is time, Ω is the angular frequency deviation from the central angular frequency *ω*_0_ and 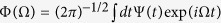. The input beam is divided into the two arms of a Michelson interferometer with the help of a polarising beam splitter (PBS_1_). Light beams with orthogonal polarisations traversing each arm of the interferometer are delayed *τ*_0_ and *τ*_0_ + *τ*, respectively, which constitute the weak coupling between the two degrees of freedom. After recombination of the two orthogonal signals in the same PBS_1_, the combination of a liquid-crystal variable retarder (LCVR) and a second polarising beam splitter (PBS_2_) performs the post-selection of the polarisation of the output state, projecting the incoming signal into the polarisation states 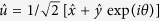 and 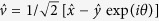. The spectral shape of the signals in the two output ports write (not normalized)


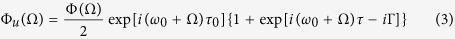



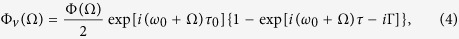


where *τ*_0_ and *τ*_0_ + *τ* are the delays and Γ = *π*/2 + *θ*.

**Figure 2:**
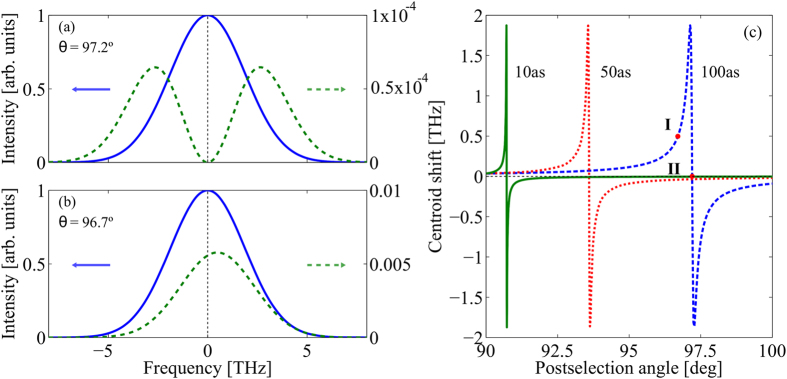
Spectrum of Φ_*u*_(Ω) measured at the corresponding output port of *PBS*_2_. (**a**,**b**) Spectral shape of the mode functions for *τ* = 0 (solid blue line) and *τ* = 100 as (dashed green line). In (**a**) the post-selection angle *θ* is 97.2^°^, so as to fulfil the condition *ω*_0_*τ* − Γ = *π*. In (**b**) the angle *θ* is 96.7^°^. (**c**) Shift of the centroid of the spectrum of the output pulse after projection into the polarisation state 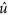 in PBS_2_, as a function of the post-selection angle *θ*. Green solid line: *τ* = 10 as; Dotted red line: *τ* = 50 as, and dashed blue line: *τ* = 100 as. Label **I** corresponds to *θ* = 96.7^°^ [mode for *τ* = 100 as shown in (**b**)]. Label **II** corresponds to *θ* = 97.2^°^, where the condition *ω*_0_*τ* − Γ = *π* is fulfiled [mode for *τ* = 100 shown in (**a**)]. It yields the minimum mode overlap between states with *τ* = 0 and *τ* ≠ 0. Data: *λ*_0_ = 1.5 *μ*m and *T*_0_ = 100 fs.

After the signal projection performed after PBS_2_, the WVA scheme distinguishes different states, corresponding to different values of the temporal delay *τ*, by measuring the spectrum of the outgoing signal in the selected output port. The different spectra obtained for delays *τ* = 0 and *τ* = 100 as, for two different polarization projections, are shown in Fig. 2(a,b). To characterize different modes one can measure the centroid shift of the spectrum of the signal in one output port as a function of the delay *τ*. If we consider the signal Φ_*u*_(Ω), the shift of the centroid is given by 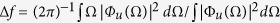. Figure 2(c) shows the centroid shift of the output signal Φ_*u*_(Ω) for *τ* ≠ 0, which reads


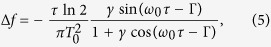


where


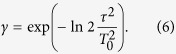


The differential power between both signals (with *τ* = 0 and *τ* ≠ 0) reads


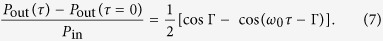


When there is no polarisation-dependent time delay (*τ* = 0), the centroid of the spectrum of the output signal is the same than the centroid of the input laser beam, i.e., there is no shift of the centroid (Δ*f* = 0). However, the presence of a small *τ* can produce a large and measurable shift of the centroid of the spectrum of the signal.

## Results

### View of weak value amplification from quantum estimation theory

Detecting the presence (*τ* ≠ 0) or absence (*τ* = 0) of a temporal delay between the two coherent orthogonally-polarised beams after recombination in PBS_1_, but before traversing PBS_2_, is equivalent to detecting which of the two quantum states,


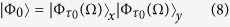


or


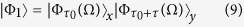


is the output quantum state which describes the coherent pulse leaving PBS_1_. (*x*, *y*) designates the corresponding polarisations. The spectral shape (mode function) Φ writes


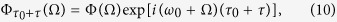


where Φ(Ω) is the spectral shape of the input coherent laser signal.

The minimum probability of error that can be made when distinguishing between two quantum states is related to the trace distance between the states^26^. For two pure states, Φ_0_ and Φ_1_, the (minimum) probability of error is^15^,^27^,^28^


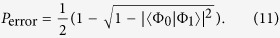


For Φ_0_ = Φ_1_, *P*_error_ = 0.5. On the contrary, to be successful in distinguishing two quantum states with low probability of error (*P*_error_ ~ 0) requires 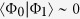, i.e., the two states should be close to orthogonal.

The coherent broadband states considered here can be generally described as single-mode quantum states where the mode is the corresponding spectral shape of the light pulse. Let us consider two single-mode coherent beams


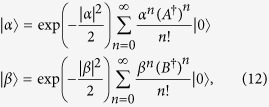


where *A* and *B* are the two modes


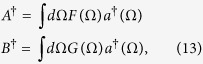


and |*α*|^2^ and |*β*|^2^ are the mean number of photons in modes *A* and *B*, respectively. The mode functions *F* and *G* are assumed to be normalized, i.e., 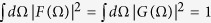. The overlap between the quantum states, 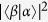, reads


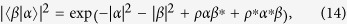


where we introduce the mode overlap *ρ* that reads


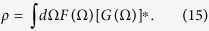


In order to obtain Eq. (14) we have made use of 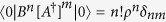. For *ρ* = 1 (coherent beams in the same mode but with possibly different mean photon numbers) we recover the well-known formula for single-mode coherent beams^29^: 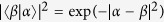.

Making use of Eqs. (10), (14) and (15) we obtain


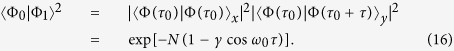


In the WVA scheme considered here, the signal after PBS_2_ is projected into the orthogonal polarisation states 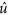 and 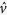, and as a result the signals in both output ports are given by Eqs. (3) and (4). Making use of Eqs. (3), (4) and (15) one obtains that the mode overlap (for Φ_*u*_) reads


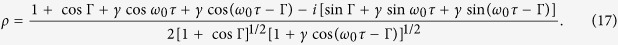


**Figure 3:**
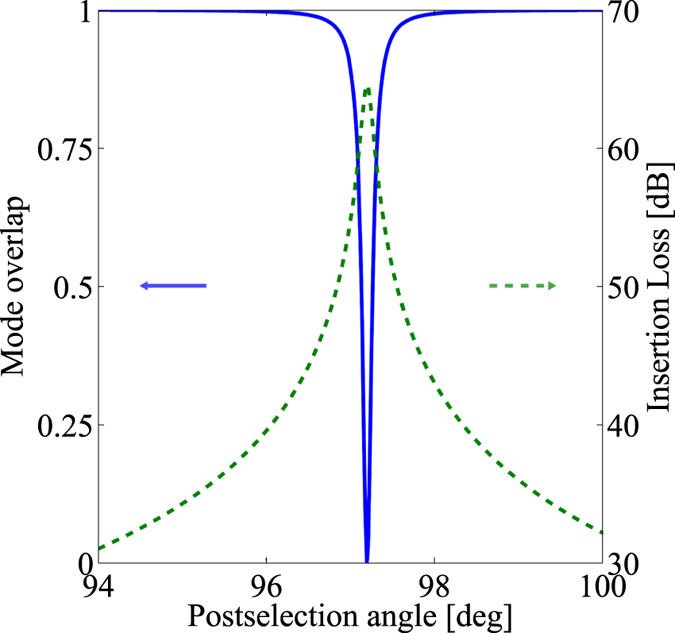
Mode overlap and insertion loss as a function of the post-selection angle. Mode overlap *ρ* of the mode functions corresponding to the quantum states with *τ* = 0 and *τ* = 100 as, as a function of the post-selection angle *θ* (solid blue line). The insertion loss, given by 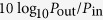 is indicated by the dotted green line. The minimum mode overlap, and maximum insertion loss, corresponds to the post-selection angle *θ* that fulfils the condition *ω*_0_*τ* − Γ = *π*, which corresponds to *θ* = 97.2^°^. Data: *λ*_0_ = 1.5 *μ*m, *T*_0_ = 100 fs.

For *τ* = 0, and therefore *γ* = 1, we obtain *ρ* = 1. Figure 3 shows the mode overlap of the signal in the corresponding output port for a delay of *τ* = 100 as. The mode overlap has a minimum for *ω*_0_*τ* − Γ = *π*, where the two mode functions becomes easily distinguishable, as shown in Fig. 2(a). The effect of the polarisation projection, a key ingredient of the WVA scheme, can be understood as a change of the mode overlap (*mode distinguishability*) between states with different delay *τ*.

However, an enhanced mode distinguishability in this output port is accompanied by a corresponding increase of the insertion loss, as it can be seen in Fig. 3. The insertion loss, *P*_out_(*τ*)/*P*_in_ = 1/2[1 + *γ*cos(*ω*_0_*τ* − Γ)], is the largest when the modes are close to orthogonal (*ρ* ~ 0). The quantum overlap between the states reads


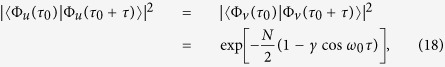


so


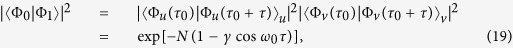


which is the same result [see Eq. (16)] obtained for the signal after PBS_1_, but before PBS_2_. Both effects indeed compensate, as it should be, since the trace distance between quantum states is preserved under unitary transformations.

We can also see the previous results from a slightly different perspective making use of quantum estimation theory (see chapter 4 in^15^). The WVA scheme considered throughout can be thought as a way of estimating the value of the single parameter *τ* with the help of a light pulse in a coherent state 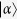. Since the quantum state is pure, the lower bound to the variance of an unbiased estimate of the parameter *τ* variance reads


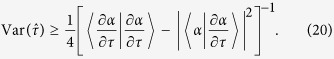


For a coherent product state of the form 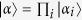, where the index *i* refers to different frequency modes, one obtains that *Q* = ∑_*i*_*Q*_*i*_, where 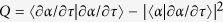 and 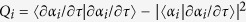. If *α*_*i*_ = *β*_*i*_exp(*iφ*_*i*_), where |*β*_*i*_|^2^ is the mean number of photons in frequency mode *i* and only *φ*_*i*_ depends on the parameter *τ* as *φ*_*i*_ = (*ω*_0_ + Ω_*i*_)*τ*, one obtains that 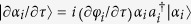, where 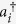 is the creation operator of the corresponding frequency mode. Making use of Eq. (9), one obtains that here the lower bound reads


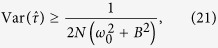


where 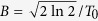 is the rms bandwidth in angular frequency of the pulse. In all cases of interest *B* ≪ *ω*_0_. This limit is a fundamental limit that set a bound to the minimum variance that any measurement can achieve. It is unchanged by unitary transformations and only depends on the quantum state considered.

Inspection of Eqs. (16) and (19) seems to indicate that a measurement after projection in any basis, the core element where it is embedded the weak amplification scheme, provides no fundamental advantage in metrology. The fact that there might be no fundamental advantage in quantum noise limited measurements for WVA, under certain general conditions, has been pointed out previously^30^, even though they also stated that alternative assumptions can also change its conclusions. Moreover, it is important to notice that this *lack of enhancement of sensitivity* is related to the fact that alternative schemes to WVA that use other characteristics of the quantum state at hand can offer better sensitivity (for instance, see an example in^31^). Notice that the result obtained implies that the only relevant factor limiting the sensitivity of detection is the quantum nature of the light used (a *coherent state* in our case). We are implicitly assuming that a) we have full access to all relevant characteristics of the output signals; and b) detectors are ideal, and can detect any change, as small as it might be, if enough signal power is used. If this is the case, weak value amplification provides no enhancement of the sensitivity.

However, these assumptions can be far from truth in many realistic experimental situations. In the laboratory, the quantum nature of light is an important factor, but not the only one, limiting the capacity to measure tiny changes of variables of interest. On the one hand, most of the times we detect only certain characteristic of the output signals, probably the most relevant, but this is still partial information about the quantum state. On the other hand, detectors are not ideal and noteworthy limitations to its performance can appear. To name a few, they might no longer work properly above a certain photon number input, electronics and signal processing of data can limit the resolution beyond what is allowed by the specific quantum nature of light, conditions in the laboratory can change randomly effectively reducing the sensitivity achievable in the experiment. Surely, all of these are *technical* rather than *fundamental* limitations, but in many situations the ultimate limit might be *technical* rather than *fundamental*. In this scenario, we show below that weak value amplification can be a *valuable* and an *easy* option to overcome all of these technical limitations, as it has been demonstrated in numerous experiments.

## Discussion

### Advantages of using weak value amplification (I): when the detector cannot work above a certain photon number

The question if WVA can be turned useful for metrology applications when considering that the amount of data is finite has been at the center of the discussion about the *true utility* of WVA schemes. In^30^ they noticed that their conclusions might not hold in this case, while in^12^ they claim that WVA is sub-optimal for any amount of data. The advantage of using WVA under the restriction of finite data is pointed out by^13^ as one of its main characteristics, and its technical advantages are also discussed in^10^. Here we quantify, making use of the concept of trace distance, how much can be gained using WVA in the implementation of WVA considered versus not using any projection at all. Again, it is important to notice that we are comparing two approaches (projection vs not projection) for a certain measurement that refers to partial information available about the quantum states. Therefore our results might not contradict claims about the existence, maybe in principle, of other experimental approaches that can equal, or even improve, what WVA can achieve.

**Figure 4:**
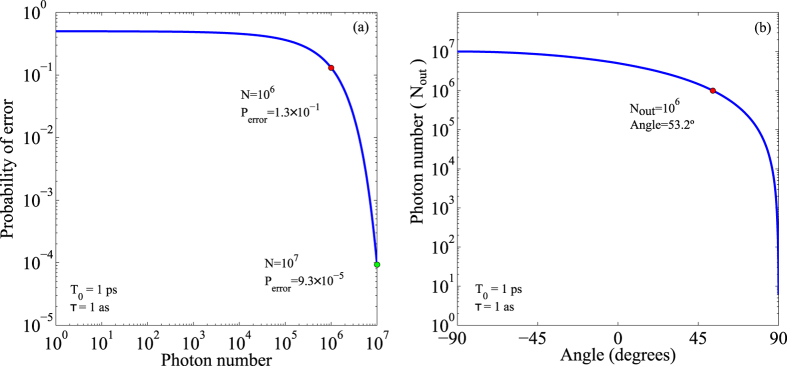
Reduction of the probability of error using a weak value amplification scheme. (**a**) Minimum probability of error as a function of the photon number *N* that leaves the interferometer. The two points highlighted corresponds to *N* = 10^6^, which yields *P*_error_ = 1.3 × 10^−1^, and *N* = 10^7^, which yields *P*_error_ = 9.3 × 10^−5^. (**b**) Number of photons (*N*_out_) after projection in the polarisation state 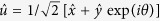, as a function of the angle *θ*. The input number of photons is *N* = 10^7^. The dot corresponds to the point *N*_out_ = 10^6^ and *θ* = 53.2^°^. Pulse width: *T*_0_ = 1 ps; temporal delay: *τ* = 1 as.

Let us suppose that we have at hand light detectors that cannot be used with more than *N*_0_ photons. Figure 4(a) shows the minimum probability of error as a function of the number of photons (*N*) entering the interferometer. For *N* = *N*_0_ = 10^6^, inspection of the figure shows that the probability of error is *P*_error_ = 1.3 × 10^−1^. This is the best we can do with this experimental scheme and these particular detectors without resorting to weak value amplification. However, if we project the output signal from the interferometer into a specific polarisation state, and increase the flux of photons, we can decrease the probability of error, without necessarily going to a regime of high depletion of the signal^18^. For instance, with *θ* = 53.2^°^, and a flux of photons of *N* = 10^7^, so that after projection *N*_out_ = *N*_0_ = 10^6^ photons reach the detector, the probability of error is decreased to *P*_error_ = 9.3 × 10^−5^, effectively enhancing the sensitivity of the experimental scheme (see Fig. 4(b)). The probability of error can be further decreased, also for other projections, at the expense of further increasing the input signal *N*.

In general, the overlap between the states, independent of any projection, is


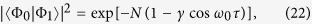


where *N* is the number of input photons. If the detectors cannot handle more than *N*_0_ photons, without a WVA scheme the input signal is limited by *N* < *N*_0_, so that the minimum overlap achievable is


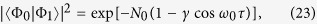


that sets a lower bound to the minimum probability of error that can be achieved in this type of measurements. However, by making use of a WVA scheme, we can select a post-selection angle Γ and increase the input photon flux so that *N*_0_ photons reach the detector. In this case we need to enhance the input number of photons to be


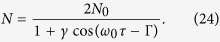


The new quantum overlap, still taking into account that only *N*_0_ can be handled by the detectors, is


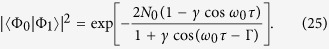


This shows that when the number of photons that the detection scheme can handle is limited, projection into a particular polarization state, at the expense of increasing the signal level, can be advantageous since the minimum probability of error achievable is decreased. From a quantum estimation point of view, WVA decreases the minimum probability of error reachable, since the projection makes possible to use the maximum number of photons available (*N*_0_) with a corresponding decrease in mode overlap. Notice that the effect of using different polarization projections can be beautifully understood as reshaping of the balance between signal level and mode overlap.

### Advantages of using weak value amplification (II): when the detector cannot differentiate between two signals

All experimental configurations show a limit on the amount of signal they can handle and how much sensitivity they can offer. On each specific experimental implementation, one or the other can be the main limiting factor that determines the tiniest change of a variable that can be measured. In many cases of interest, where the amount of signal can be safely increased, technical characteristics of the measuring apparatus establish a lower bound on the level of resolution achievable in the experiment. As a typical example, the experimental arrangement used in^20^ to demonstrate the WVA scheme considered throughout this paper, it could no detect shifts of the centroid of any spectral distribution below *δλ* ~ 0.01 nm. On practical terms, one can increase the signal to obtain a better resolution for *δλ* ≥ 0.01 nm, while this was not feasible for *δλ* ≤ 0.01 nm.

To be more specific, let us consider that specific experimental conditions makes hard, even impossible, to detect very similar modes, i.e., with mode overlap *ρ* ~ 1. We can represent this by assuming that there is an *effective* mode overlap (*ρ*_eff_) which takes into account all relevant experimental limitations of a specific set-up, given by


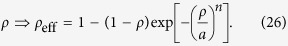


**Figure 5:**
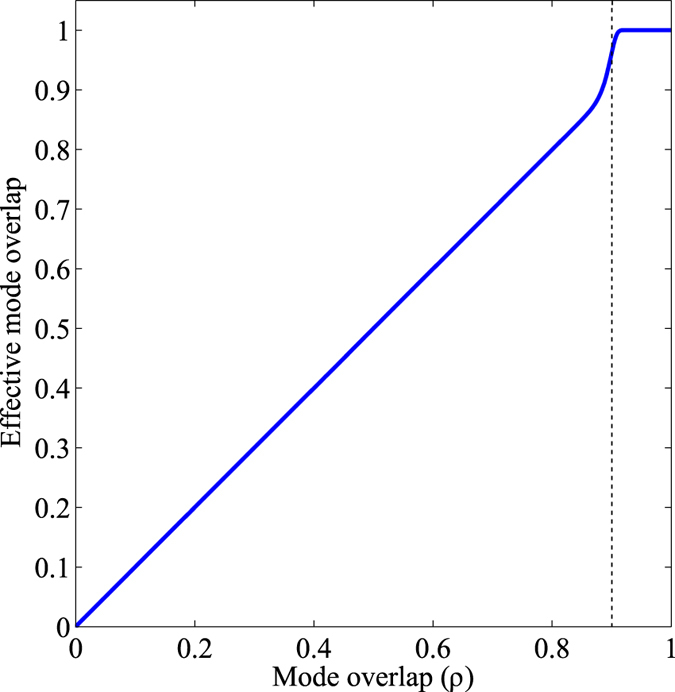
Effective mode overlap. For *ρ* > 0.9 the detection system cannot distinguish the states of interest. Data: *a* = 0.9 and *n* = 100.

Figure 5 shows an example where we assume that detected signals corresponding to *ρ* > 0.9 cannot be safely distinguished due to technical restrictions of the detection system. For *ρ* > 0.9, *ρ*_eff_ = 1, so the detection system cannot distinguish the states of interest even by increasing the level of the signal. On the contrary, for smaller values of *ρ*, accessible making use of a weak amplification scheme, this limitation does not exist since the detection system can resolve this modes when enough signal is present.

### Advantages of using weak value amplification (III): enhancement of the Fisher information

Up to now, we have used the concept of trace distance to look for the minimum probability of error achievable in *any* measurement when using a given quantum state. In doing that, we only considered how the quantum state changes for different values of the variable to be measured, without any consideration of how this quantum state is going to be detected. If we would like to include in the analysis additional characteristics of the detection scheme, one can use the concept of Fisher information, that requires to consider the probability distribution of possible experimental outcomes for a given value of the variable of interest. In this approach, one chooses different probability distributions to describe formally *characteristics* of specific detection scheme^10^.

Let us assume that to estimate the value of the delay *τ*, we measure the shift of the centroid (Δ*f*) of the spectrum Φ_*u*_(Ω), given by Eq. (4). A particular detection scheme will obtain a set of results {(Δ*f*)_*i*_}, *i* = 1..*M* for a given delay *τ. M* is the number of photons detected. The Fisher information *I*(*τ*) provides a bound of 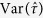 for any unbiased estimator when the probability distribution *p*({(Δ*f*)_*i*_}|*τ*) of obtaining the set {(Δ*f*)_*i*_}, for a given *τ*, is known.

In general, to determine the value of a parameter of interest *τ*, we perform repeated measurements to estimate its value. From the measurements we obtain a distribution of outcomes {*x*} which can be characterized by a probability distribution *p*(*x*|*τ*) that depends on the value of *τ*. The variance of any unbiased estimator that makes use of the ensemble {*x*} is bounded from below by 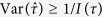, where the Fisher information *I*(*τ*) reads 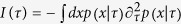. When the Fisher function can be written as *I*[*η*(*τ*)], where *η* is the variable that we measure, the Fisher information can be written as 
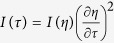
. If we assume that the probability distribution *p*({(Δ*f*)_*i*_}|*τ*) is Gaussian, with mean value Δ*f* given by Eq. (5) and variance *σ*^2^, determined by the errors inherent to the detection process, the Fisher information reads


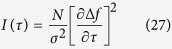


where


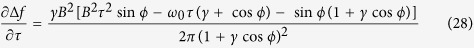


and *ϕ* = *ω*_0_*τ* − Γ.

For *ϕ* = 0, i.e., the angle of post-selection is *θ* = −*π*/2 + *ω*_0_*τ*, the flux of photons detected is *N* = (*N*_0_/2)(1 + *γ*) and the Fisher information is


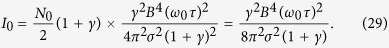


Notice that *θ* = −*π*/2 corresponds to considering equal input and output polarization state, i.e., no weak value amplification scheme. For *ϕ* = *π*, where the angle of post-selection is *θ* = *π*/2 + *ω*_0_*τ*, the flux of photons detected is *N* = (*N*_0_/2)(1 − *γ*), and we have


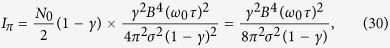


where *θ* = *π*/2 corresponds to considering an output polarisation state orthogonal to the input polarisation state i.e., when the effect of weak value amplification is most dramatic, as it can be easily observed in Fig. 2(a). The Fisher bound for Φ = *π* is a factor *I*_*π*_/*I*_0_ = (1 + *γ*)/(1 − *γ*) larger than the bound for Φ = 0, so WVA achieves enhancement of the Fisher information. This Fisher information enhancement effect, which does not happen always, has been observed for certain WVA schemes^10^,^32^. We should note that the enhancement of the Fisher information obtained comes from comparing two different and specific measurements, the results of projecting the signal that bears the sought-after information in different states in each case. Since we are not considering more general measurements to obtain the optimum measurement that maximize the Fisher information, it might be possible that other measurement schemes could further increase the Fisher information.

There is no contradiction between the facts that the minimum probability of error, obtained by making use of the concept of trace distance, is not enhanced by WVA, while at the same time there can be enhancement of the Fisher information with a particular measurement setup. By selecting a particular probability distribution to evaluate the Fisher information, we include information about the detection scheme. In our case, we estimate the value of *τ* by measuring the *τ*-dependent shift of the centroid of the spectrum of the signal in one output port after PBS_2_, which is only part of all the information available, given by the full signal in Eqs. (3) and (4). We also assumed a Gaussian probability distribution with a constant variance *σ*^2^ independent of *τ*. The minimum probability of error that was obtained making use of the trace distance depends on the full information available (the quantum state) before any particular detection. An unitary transformation, as the one where WVA is embedded, does not modify the bound. On the contrary, the Fisher information, by using a particular probability distribution to describe the possible outcomes in an particular experiment, selects certain aspects of the quantum state to be measured (*partial information*), and this bound can change in a WVA scheme, although the bound should be obviously always above the one determined by the minimum probability of error. In this restrictive scenario, the use of certain polarization projections can be preferable.

The existence and nature of these different bounds might possibly explain certain confusion about the capabilities of WVA, whether WVA is considered to provide any metrological advantage or not. On the one hand, if we consider the trace distance, or the quantum Cramér-Rao inequality, without any consideration about how the quantum states are detected, post-selection inherent in WVA does not lower the minimum probability of error achievable, so from this point of view WVA offers no metrological advantage. On the other hand, in certain scenarios, the Fisher information, when it takes into account *information about the detection scheme*, can be enhanced due to post-selection. In this sense, one can think of WVA as an advantageous way to optimize a particular detection scheme.

## Conclusions

WVA is embedded into measurement schemes that makes use of linear optics unitary transformations. Therefore, if the only limitations in a measurement are due to the quantum nature (*intrinsic statistics*) of the light, for instance, the presence of shot noise in the case of coherent beams, WVA does not offer any advantage regarding any decrease of the minimum probability of error achievable. This is shown by making use of the trace distance between quantum states, which set sensitivity bounds that are independent of any particular post-selection. However, notice that this implicitly assume that full information about the quantum states used can be made available, and detectors are ideal, so they can detect any change of the variable of interest, as small as it might be, provided there is enough signal power. For instance, here we decided to measure the centroid shift of the spectral shape (specific information) in a given projection (we neglected all information concerning the other orthogonal projection. Therefore WVA cannot do better than using the full information contained in the quantum state.

Nevertheless, these assumptions are in many situations of interest far from true. These limitations, sometimes refereed as *technical noise*, even though not fundamental (one can always imagine using a better detector or a different detection scheme) are nonetheless important, since they limit the accuracy of specific detection systems at hand. In these scenarios, the importance of weak value amplification is that by decreasing the mode overlap associated with the states to be measured and possibly increasing the intensity of the signal, the weak value amplification scheme allows, in principle, to distinguish them with lower probability of error.

We have explored some of these scenarios from an quantum estimation theory point of view. For instance, we have seen that when the number of photons usable in the measurement is limited, the minimum probability of error achievable can be effectively decreased with weak value amplification. We have also analyzed how weak value amplification can differentiate between *in practice*-indistinguishable states by decreasing the mode overlap between its corresponding mode functions.

Finally we have discussed how the confusion about the usefulness of weak value amplification can possibly derive from considering different bounds related to how much sensitivity can, in principle, be achieved when estimating a certain variable of interest. One might possibly say that the advantages of WVA *have nothing to do with fundamental limits and should not be viewed as addressing fundamental questions of quantum mechanics*^33^. However, *from a practical rather than fundamental point of view*, the use of WVA can be advantageous in experiments where sensitivity is limited by experimental (technical), rather than fundamental, uncertainties. In any case, if a certain measurement is *optimum* depends on its capability to effectively reach any bound that might exist.

## Additional Information

**How to cite this article**: Torres, J. P. and Salazar-Serrano, L. J. Weak value amplification: a view from quantum estimation theory that highlights what it is and what isn’t. *Sci. Rep.* **6**, 19702; doi: 10.1038/srep19702 (2016).

## References

[b1] Aharonov Y., Albert D. Z. & Vaidman L. How the result of a measurement of a component of the spin of a spin-1/2 particle can turn out to be 100. Phys. Rev. Lett. 60, 1351–1354 (1988). 10038016 10.1103/PhysRevLett.60.1351

[b2] Hosten O. & Kwiat P. Observation of the spin Hall effect of light via weak measurements. Science 319, 787–790 (2008). 18187623 10.1126/science.1152697

[b3] Zhou X., Zhou, Ling X., Luo H. & Wen S. Identifying graphene layers via spin Hall effect of light. App. Phys. Lett. 101, 251602 (2012).

[b4] Ben Dixon P., Starling D. J., Jordan A. N. & Howell J. C. Ultrasensitive beam deflection measurement via interferometric weak value amplification. Phys. Rev. Lett. 102, 173601 (2009). 19518781 10.1103/PhysRevLett.102.173601

[b5] Pfeifer M. & Fischer P. Weak value amplified optical activity measurements. Opt. Express 19, 16508–16517 (2011). 21935015 10.1364/OE.19.016508

[b6] Howell J. C., Starling D. J., Dixon P. B., Vudyasetu K. P. & Jordan A. N. Precision frequency measurements with interferometric weak values. Phys. Rev. A 82, 063822 (2010).

[b7] Egan P. & Stone J. A. Weak-value thermostat with 0.2 mK precision. Opt. Lett. 37, 4991–4993 (2012). 23202114 10.1364/OL.37.004991

[b8] Xu X. Y. *et al.* Phase estimation with weak measurement using a white light source. Phys. Rev. Lett. 111, 033604 (2013). 23909319 10.1103/PhysRevLett.111.033604

[b9] Dressel J., Malik M., Miatto F. M., Jordan A. N. & Boyd R. W. Colloquium: Understanding quantum weak values: Basics and applications. Rev. Mod. Phys. 86, 307–316 (2014).

[b10] Jordan A. N., Martíez-Rincón J. & Howell J. C. Technical Advantages for Weak-Value Amplification: When Less Is More. Phys. Rev. X 4, 011031 (2014).

[b11] Knee G. C. & Gauger E. M. When Amplification with Weak Values Fails to Suppress Technical Noise. Phys. Rev. X 4, 011032 (2014).

[b12] Ferrie C. & Combes J. Weak Value Amplification is Suboptimal for Estimation and Detection. Phys. Rev. Lett. 112, 040406 (2014). 24580424 10.1103/PhysRevLett.112.040406

[b13] Vaidman L. Comment on *Weak value amplification is sub-optimal for estimation and detection*. Phys. Rev. Lett. 111, 033604 (2013). arXiv:1402.0199v1 [quant-ph] (2014)(date of access: 20/10/2015).23909319

[b14] Knee G. C., Combes J., Christopher Ferrie C. & Gauger E. M. Weak-value amplification: state of play. arXiv:1410.6252 [quant-ph] (2014) (date of access: 20/10/2015).

[b15] Helstrom C. W. Quantum Detection and Estimation Theory Ch. 8, 235–292 (Academic press Inc., New York, 1976).

[b16] Nielsen M. A. & Chuang I. L. Quantum computation and quantum information, Ch. 9, 399–416, (Cambridge University Press, Cambridge, 2000).

[b17] Zhang L., Datta A. & Walsmely I. A. Precision metrology using weak measurements. Phys. Rev. Lett. 114, 210801 (2015). 26066422 10.1103/PhysRevLett.114.210801

[b18] Torres J. P., Puentes G., Hermosa N. & Salazar-Serrano L. J. Weak interference in the high-signal regime. Opt. Express 20, 18869–18875 (2012). 23038526 10.1364/OE.20.018869

[b19] Brunner N. & Simon C. Measuring small longitudinal phase shifts: weak measurements of standard interferometry. Phys. Rev. Lett. 105, 010405 (2010). 20867428 10.1103/PhysRevLett.105.010405

[b20] Salazar-Serrano L. J., Janner D., Brunner N., Pruneri V. & Torres J. P. Measurement of sub-pulse-width temporal delays via spectral interference induced by weak value amplification. Phys. Rev. A 89, 012126 (2014).

[b21] Duck I. M., Stevenson P. M. & Sudarhshan E. C. G. The sense in which a weak measurement of a spin-1/2 particles’s spin component yields a value of 100. Phys. Rev. D 40, 2112–2117 (1989). 10.1103/physrevd.40.2112 10012041

[b22] Howell J. C., Starling D. J., Dixon P. B., Vudyasetu K. P. & Jordan A. N. Interferometric weak value deflections: quantum and classical treatments. Phys. Rev. A 81, 033813 (2010).

[b23] Ritchie N. W., Story J. G. & Hulet R. G. Realization of a measuremernt of a weak value. Phys. Rev. Lett. 66, 1107–1110 (1991). 10043997 10.1103/PhysRevLett.66.1107

[b24] Strubi G. & Bruder C. Measuring Ultrasmall Time Delays of Light by Joint Weak Measurements. Phys. Rev. Lett. 110, 083605 (2012). 23473146 10.1103/PhysRevLett.110.083605

[b25] Li C.-F. *et al.* Ultrasensitive phase estimation with white light. Phys. Rev. A 83, 044102 (2011).

[b26] Fuchs C. A. & van de Graaf J. Cryptographic Distinguishability Measures for Quantum Mechanical States. IEEE T. Inform. Theory 45, 1216–1227 (1999).

[b27] Englert B.-G. Fringe Visibility and Which-Way Information: An Inequality,” Phys. Rev. Lett. 77, 2154–2157 (1996). 10061872 10.1103/PhysRevLett.77.2154

[b28] Ou Z. Y. Complementarity and Fundamental Limit in Precision Phase Measurement. Phys. Rev. Lett. 77, 2352–2355 (1996). 10061932 10.1103/PhysRevLett.77.2352

[b29] Glauber R. J. Coherent and incoherent states of the radiation field. Phys. Rev. 131, 2766–2788 (1966).

[b30] Tanaka S. & Yamamoto N. Information amplification via postselection: a parameter-estimation study, Phys. Rev. A 88, 042116 (2013).

[b31] Knee G. & Munro W. J. Fisher information versus signal-to-noise ratio for a split detector. Phys. Rev. A 92, 012130 (2015).

[b32] Viza G. I. *et al.* Weak-values technique for velocity measurements. Opt. Lett. 38, 2949–2952 (2013). 24104618 10.1364/OL.38.002949

[b33] Combes J., Ferrie C., Zhang J., Carlton M. & Caves C. M. Quantum limits on postselected, probabilistic quantum metrology. Phys. Rev. A 89, 052117 (2014).

